# On the Effectiveness of Impedance-Based Fingerprint Presentation Attack Detection

**DOI:** 10.3390/s21175686

**Published:** 2021-08-24

**Authors:** Jascha Kolberg, Daniel Gläsner, Ralph Breithaupt, Marta Gomez-Barrero, Jörg Reinhold, Arndt von Twickel, Christoph Busch

**Affiliations:** 1 da/sec—Biometrics and Internet Security Research Group, Hochschule Darmstadt, 64295 Darmstadt, Germany; d.glaesner@id-loop.de (D.G.); christoph.busch@h-da.de (C.B.); 2 Federal Office for Information Security, 53133 Bonn, Germany; ralph.breithaupt@bsi.bund.de (R.B.); arndt.twickel@bsi.bund.de (A.v.T.); 3 Fakultät Wirtschaft, Hochschule Ansbach, 91522 Ansbach, Germany; marta.gomez-barrero@hs-ansbach.de; 4 Jenetric GmbH, 07745 Jena, Germany; j.reinhold@id-loop.de

**Keywords:** fingerprint recognition, presentation attack detection, impedance measurement, unknown attacks

## Abstract

Within the last few decades, the need for subject authentication has grown steadily, and biometric recognition technology has been established as a reliable alternative to passwords and tokens, offering automatic decisions. However, as unsupervised processes, biometric systems are vulnerable to presentation attacks targeting the capture devices, where presentation attack instruments (PAI) instead of bona fide characteristics are presented. Due to the capture devices being exposed to the public, any person could potentially execute such attacks. In this work, a fingerprint capture device based on thin film transistor (TFT) technology has been modified to additionally acquire the impedances of the presented fingers. Since the conductance of human skin differs from artificial PAIs, those impedance values were used to train a presentation attack detection (PAD) algorithm. Based on a dataset comprising 42 different PAI species, the results showed remarkable performance in detecting most attack presentations with an APCER = 2.89% in a user-friendly scenario specified by a BPCER = 0.2%. However, additional experiments utilising unknown attacks revealed a weakness towards particular PAI species.

## 1. Introduction

Within the last decade, biometric recognition systems have been deployed in several applications used in our daily lives. Almost all new smartphones can be unlocked with biometrics, which is very user-friendly. On the other hand, border control utilises biometrics to enhance the security. Hence, the usage of biometric recognition itself is flexible and suits different scenarios. In this context, the fingerprint has always been one of the most used biometric characteristics [1].

However, with the capture devices being exposed to the public, biometric systems are vulnerable to external attacks [2]. This kind of attack is defined within the ISO/IEC 30107-1 [3] as a “presentation to the biometric data capture subsystem with the goal of interfering with the operation of the biometric system”. The intention of the attacker can be either to impersonate someone’s identity or to conceal his own identity. In both cases, a presentation attack instrument (PAI) instead of the bona fide characteristic is presented to the capture device. In this context, a group of PAIs made from the same material is called a PAI species. As a consequence, a secure biometric system requires an automated presentation attack detection (PAD) module, which needs to learn the differences between bona fide presentations (BPs) and attack presentations (APs) [4]. Correct assignment of the two classes is becoming more complicated with the many options available for creating PAIs. For fingerprint artefacts in particular, there are multiple recipes based on a variety of materials available [5].

The challenge of PAD was addressed by multiple international research projects, such as Tabula Rasa [6], BEAT [7], Odin [8], and RESPECT [9]. Additionally, the LivDet challenges [10,11] have invited researchers from academia and industry to benchmark their PAD algorithms on identical datasets since 2009. These efforts allowed significant research and development of new countermeasures across different biometric characteristics. In general, PAD methods can be categorised into software-based and hardware-based approaches. While the first category analyses the samples acquired with legacy fingerprint captures in a deeper way (i.e., PAD based solely on data of the biometric sensor), the latter one introduces additional sensors to capture complementary information solely used by the PAD algorithm (i.e., PAD based on data of additional dedicated PAD sensors). Examples of hardware-based methods are multispectral illuminations and pulse detection, which require additional modules and thus usually result in bigger capture devices than pure fingerprint sensors [12].

This work focused on adding a hardware-based sensor to an existing capture device and validating whether this additional information is suited to detecting APs for fingerprint recognition. The idea is that human skin has different conductivity than the artificial materials of PAIs, and hence can be discriminated from them. Therefore, this study aims to answer the question of whether a finger’s impedance is an effective feature for reliable fingerprint PAD. To that end, we describe the functionality of the capture device which was used to acquire a dataset of 757 BPs and 915 APs from 42 different PAI species. A fingerprint PAD algorithm was trained and evaluated on this dataset.

The development of fingerprint PAD methods requires a dataset with BPs and APs. As a consequence, PAIs need to be created from either cooperative target subjects or latent (or synthetic) fingerprints. In a cooperative approach, the bona fide finger is placed in a moulding material, which includes the negative fingerprint pattern after hardening. Subsequently, this mould can be filled with the casting material (e.g., latex or ecoflex) to retrieve the actual PAI. Depending on the properties of the moulding and casting materials, the mould can be reused for additional fabrications. For latent fingerprints, the representation needs to be digitised first. Then the negative image is used to create the mould (e.g., 3D printer or laser cutter), which can be filled again with the casting material.

The remainder of this article is structured as follows: Section 2 reviews the state-of-the-art for fingerprint PAD. Our capture device is introduced in Section 3, followed by the method for fingerprint PAD in Section 4. Subsequently, the experimental evaluation is presented and discussed in Section 5, and finally, Section 6 concludes the findings.

## 2. Related Work

The PAD approaches reviewed in the following are also summarised in Table 1. Since this work proposes a new hardware-based approach and presents a detailed analysis with regard to generalisation capabilities towards unknown attacks, related work has been selected from these areas as well. Due to the fact that most of these works were tested on different datasets and report results from multiple experiments, a fair comparison is not possible. Finally, this overview is by no means complete but focuses on selected approaches to put our own contribution into context. However, the interested reader is referred to more extensive surveys on fingerprint PAD methods [13,14,15,16,17].

The publicly available LivDet datasets [18,19,20,21,22,23] established a well known and commonly used foundation for software-based fingerprint PAD development. While early fingerprint PAD algorithms mostly utilised handcrafted feature extractions and classifiers [24,25,26,27,28,29], a shift towards deep learning approaches is noticeable in more recent publications [30,31,32,33]. However, when focussing on unknown attacks and cross-sensor and cross-database scenarios, it is clear that handcrafted methods are able to outperform deep learning approaches, as was shown by the winner of the LivDet 2019 competition [34]. This work was further extended in [35] to include even more features into the fisher vector encoding before classifying these with a support vector machine (SVM).

**Table 1:** A summary of related fingerprint PAD approaches.

Year	Ref.	Approach	Description	Database (# PAI Species)
2008	[36]	handcrafted	pulse + oxygen level	own DB (1)
2011	[24]	handcrafted	static + intensity features	LivDet 2009 (3)
	[37]	handcrafted	pulse + multi-spectral	own DB (4)
2013	[38]	handcrafted	optical methods	own DB (N/A)
2014	[25]	handcrafted	data augmentation + SVM	LivDet 2009–2013 (8)
2015	[26]	handcrafted	one-class SVM	LivDet 2011 (7)
	[27]	handcrafted	one-class SVM/GMM	LivDet 2013 (7)
	[30]	deep learning	deep representations	LivDet 2013 (7)
2016	[28]	handcrafted	one-class SVMs	LivDet 2011 (7)
	[31]	deep learning	deep belief network	LivDet 2013 (7)
	[32]	deep learning	pre-trained CNNs	LivDet 2009–2013 (8)
2017	[29]	handcrafted	feature fusion	LivDet 2009–2013 (8)
	[33]	deep learning	patch-based CNN	LivDet 2011–2013 (8)
	[39]	sensor design	ultrasonic fingerprint	no DB (0)
2018	[40]	deep learning	Fingerprint Spoof Buster	LivDet 2011–2015, (12)
				MSU-FPAD, PBSKD
2019	[41]	deep learning	one-class GANs	own DB (12)
2020	[42]	handcrafted	finger vein skeleton	own DB (32)
	[43]	deep learning	adversarial learning	LivDet 2015 (9)
	[44]	deep learning	universal material generator	LivDet 2017, (12)
				MSU-FPAD, PBSKD
	[45]	deep learning	adversarial representations	LivDet 2015–2017, (11)
				MSU-FPAD
2021	[34]	handcrafted	local feature encoding	LivDet 2011–2019 (15)
	[35]	handcrafted	fisher vector encoding	LivDet 2011–2017 (13)
	[46]	deep learning	convolutional autoencoder	own DB ${}^{1}$ (45)
	[47]	deep learning	LOO benchmark	own DB ${}^{1}$ (45)
	[48]	deep learning	OCT autoencoder	own DB (101)
	[49]	sensor design	ultrasonic fingerprint	no DB (0)

${}^{1}$ Part of the dataset has already been released: https://github.com/ISICV/PADISI_USC_Dataset accessed on 20 August 2021.

In the area of deep learning, Chugh and Jain [40] proposed their Fingerprint Spoof Buster as a patch-based convolutional neural network (CNN) together with two datasets, MSU-FPAD and PBSKD. Using a training set, this approach was able to detect additional unknown attacks due to their similarity to known attacks. The authors followed up on this and extended the approach with a synthetic sample generator [44]. Based on these additional samples for different PAI species, the network could be trained on a larger dataset. Subsequently, Grosz et al. [45] combined this synthetic generator with adversarial representation learning to overcome the weakness regarding cross-sensor and unknown attack scenarios. Using an adversarial and transfer learning approach, Pereira et al. [43] improved the robustness of the model to unknown PAI species. After their encoder mapped the input to a latent space, a classifier distinguished between APs and BPs. However, another classifier additionally tried to determine the corresponding PAI species of the latent feature vector. This information was then returned to the encoder with the goal of finding a new representation that was independent of the PAI species. Thus, the encoder learnt a generalising representation that was robust to unknown PAI species.

While the previous approaches targeted the problem of unknown attacks, they still needed numerous APs for training. In the area of anomaly detection, one-class classifiers can be trained on BPs only. Hence, all attacks are unknown by default, and every presentation that appears different to the BPs in training is automatically classified as AP. In this context, some research evaluated handcrafted classifiers such as SVMs or Gaussian mixture models (GMMs), which did not see APs during training [26,27]. Later, Engelsma and Jain [41] utilised one-class generative adversarial networks (GANs) for fingerprint PAD based on images captured with the *RaspiReader* [50]. In another approach, Kolberg et al. [46] proposed a convolutional autoencoder for multi-spectral images from a camera-based capture device. In a similar fashion, Liu et al. [48] evaluated an autoencoder on samples acquired with an optical coherence tomography (OCT) sensor.

Regarding hardware-based approaches, various methods and sensing techniques have been proposed. One of the early approaches [36] combines pulse measurement with haemoglobin observation within the finger veins. Using a near infra-red light source, the oxygen saturation is analysed, which also allows the detection of cadaver fingers. The idea of extracting the finger vein skeleton for fingerprint PAD was later evaluated in [42]. The analysis showed that full fake fingers were easily detected, and for thin and transparent overlays the veins were still visible. Hence, those PAI species could not be distinguished from BPs. Following up on pulse measurement, Hengfoss et al. [37] additionally acquired multi-spectral samples. Their conclusion states that the capture time for pulse measurement exceeds the time taken for others, and thus is less favourable. A similar observation was made by Drahansky et al. [38], who tested optical methods for pulse, pressure, and skin reflections.

In a different approach, Jiang et al. [39] proposed a new ultrasonic fingerprint capture device. This technology allows one to acquire fingerprint images beneath the epidermis, which is theoretically harder to attack than, e.g., capacitive sensors. A more recent approach [49] additionally captures the finger vessels underneath the fingerprint, which in theory is even more robust against APs. However, both works did not collect APs to confirm the PAD capabilities. On the other hand, Kolberg et al. [47] presented an extensive benchmark of multiple fingerprint PAD algorithms for multi-spectral images. The leave-one-out (LOO) experiments showed that a fusion of complementary input data benefits the PAD performance in the presence of unknown attacks.

## 3. Capture Device and Data

Even if it is possible to implement software-based PAD methods to distinguish between BPs and APs, this work instead introduces a hardware-based adjustment to derive the decision based on additional data. The goal is to measure the impedance, since the conductivity of human skin differs from the conductivity other artificial materials.

**Figure 1:**
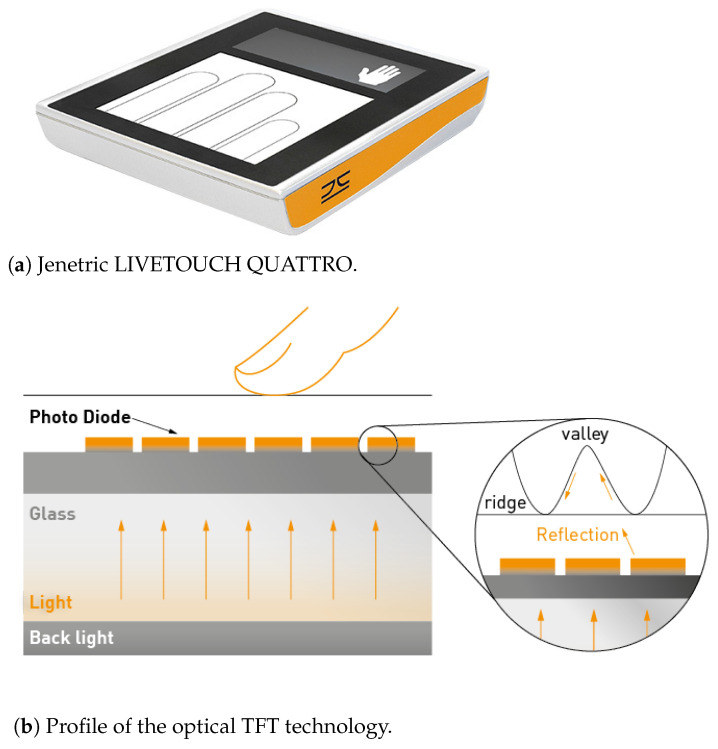
An illustration of the fingerprint capture device. ©Jenetric GmbH.

**Figure 2:**
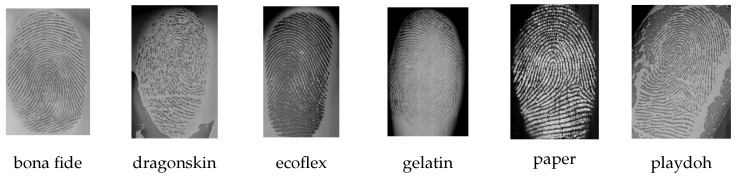
A bona fide fingerprint and five different PAIs.

The capture device is based on the Jenetric LIVETOUCH QUATTRO (https://www.jenetric.com/en/products/livetouch-quattro.html, accessed on 20 August 2021), which is depicted in Figure 1a. The optical thin film transistor (TFT) technology allows capturing up to four fingerprints at a time. In this process, the display beneath serves as illumination source (Figure 1b) and can be used for user guidance as well. While this technique enables reliable acquisitions of bona fide fingerprints, it also captures the fingerprint patterns from particular APs as shown in Figure 2. The main advantage of TFT sensors is their size. While it is possible to cover large areas (e.g., four fingers simultaneously), the glass is only 0.7 mm thick, and thus it is easy to include in various devices. As this offers huge potential for numerous applications, fingerprint PAD for TFT capture devices has relied so far on software-based analysis of fingerprint images. However, research has shown that APs can indeed have similar fingerprint quality as BPs [51], which can be a problem for purely software-based PAD solutions. On the other hand, adding established PAD sensors is difficult to do without losing the advantage of the TFT technology.

**Figure 3:**
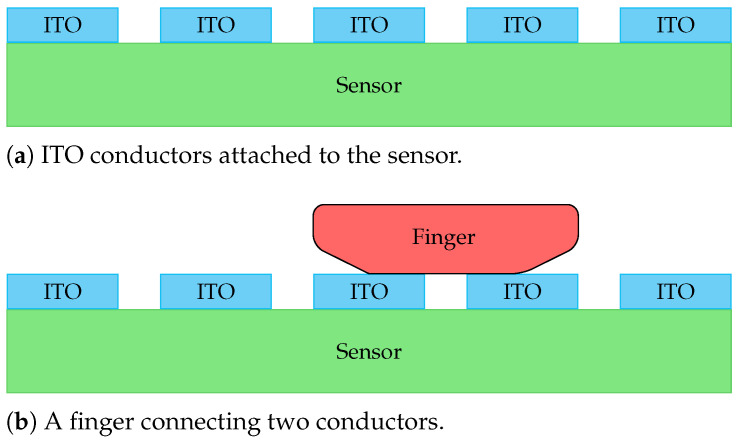
The ITO conductors are structured vertically on the capture device to lie in the same direction as the fingers.

In the proposed approach, we measure the impedance on top of the optical fingerprint sensor. To that end, the usage of transparent electrodes is necessary in order not to interfere with the fingerprint acquisition. Hence, a strip-shaped indium-tin-oxide (ITO) coating is applied to the top layer of the capture device. The electric circuit utilises an alternating current source with various specified frequencies and a peak-to-peak voltage of 1 V. Once a finger connects the two electrodes, the finger’s impedance can be measured. This PAD adjustment is illustrated in Figure 3. According to Ohm’s law, voltage drops within the circuit are indirectly proportional to the corresponding resistances. In our case, the resistance of human skin is a complex quantity, as it is composed of capacitive and ohmic resistances. Hence, the resistance of skin decreases with increasing frequency, which causes the voltage to increase.

The prototype has a controllable output, and for the context of fingerprint PAD, nine frequencies in the range between 1 and 500 kHz are used. Finally, a second-order high-pass filter in combination with a bridge rectifier removes 50 Hz noise and smoothes the signal, which thus corresponds to a DC voltage. Those measured impedance values can then be used by PAD algorithms.

## 4. Presentation Attack Detection Method

With our fingerprint PAD approach, we aimed to keep things as simple as possible, while preserving full compatibility with the base fingerprint sensor. The finger’s impedance is measured at nine selected frequencies in the range from 1 to 500 kHz, which are listed in Table 2. The first frequency was chosen to have enough distance from the 50 Hz high-pass filter to avoid interference with the attenuation. Additionally, the frequency generator is technically capped at 1 MHz; thus, the remaining frequencies were selected such that the maximum possible range is covered with few selected measurement points, while also taking into account the decreasing resistance for increasing frequencies. Therefore, for each presentation, nine float values of PAD data are acquired. No conductivity results in a zero vector and the measured impedance values rise for conductive presentations. Hence, this nine-dimensional feature vector can directly be used for classification without further pre-processing.

**Figure 4:**
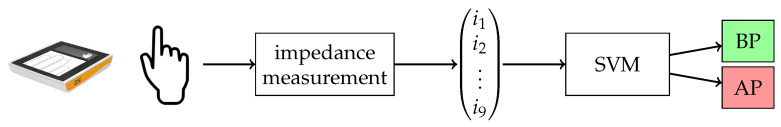
While capturing the fingerprint, the impedance is measured at nine selected frequencies. The resulting feature vector $i=\left(i_{1},\;i_{2},\;\cdots ,\;i_{9}\right)$ is processed by the SVM to derive a decision.

For this purpose, the SVM [52] is used as a classifier, since it constantly achieved remarkable performances across different fingerprint PAD studies [34,35,53,54,55,56,57]. The SVM is designed to work on high-dimensional input data and derive binary decisions by defining a hyperplane that separates both classes. Hence, it is perfectly suited for fingerprint PAD tasks. We are only interested in detecting whether an input is an AP and not which material was used to fabricate this particular PAI. In the training process, 5-fold cross-validation was used to automatically determine the best-suited hyperparameters for the RBF kernel on the available training data. During prediction, the test sample is mapped into the SVM feature space, where it can be compared to the hyperplane in order to retrieve a real-time classification result. The full fingerprint PAD processing pipeline is illustrated in Figure 4. Although the main contribution is the impedance measurement and not a classifier benchmark, the SVM approach is shown in comparison to k-nearest neighbours (KNN) and multi-layer perceptron (MLP) classifiers.

## 5. Experimental Evaluation

In this section, we first introduce the database and experimental protocol, which were used to run the experiments with the goal of answering the question of whether the impedance-based technology presented here is suited for fingerprint PAD. We then present and discuss the results.

### 5.1. Database and Experimental Protocol

**Figure 5:**
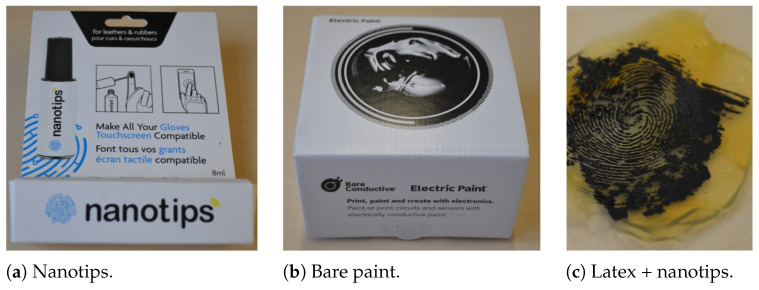
Conductive augmentations can be created by applying, e.g., nanotips (**a**) or electric bare paint (**b**) to the fingerprint PAI (**c**).

**Figure A1:**
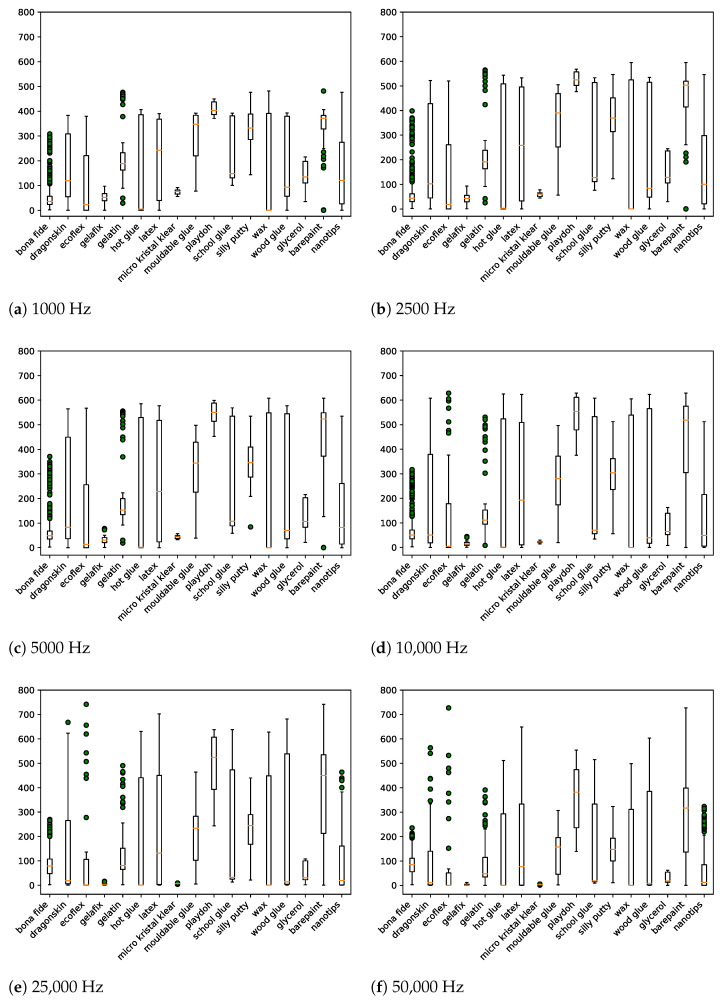

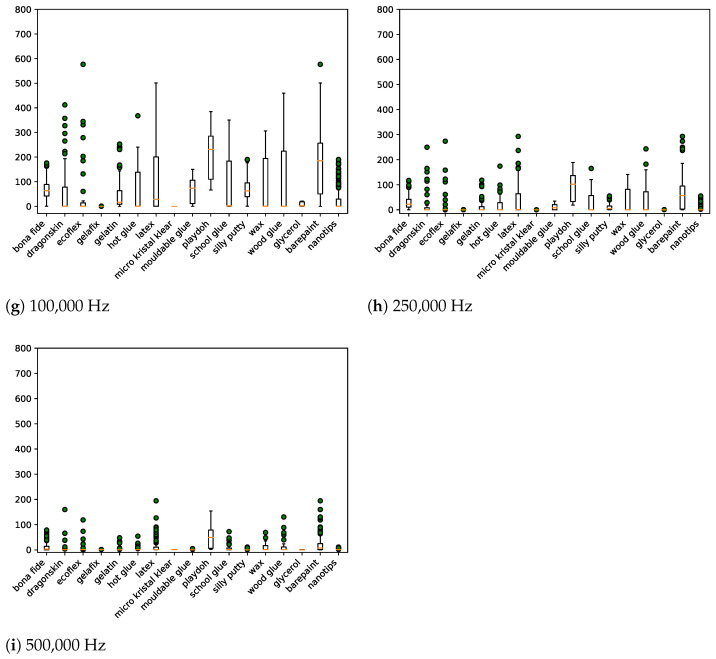
For each frequency (**a**–**i**), the plots visualise the impedance values using the median (orange), ±25% (box), the standard deviation (end of line), and outliers (green dots) for all conductive presentations.

The data acquisition was split over three distinct locations. Each party collected BPs from all ten fingers, and in total, 59 subjects contributed 757 BPs. Since the data collection took place during a pandemic, fingers and the sensor surface were disinfected between collecting data from different subjects. Apart from that, the presented fingers had different moisture levels. In addition, each party created their own PAIs, which resulted in a total of 915 APs from 42 different PAI species for the dataset. On the other hand, this led to unequal numbers of APs per PAI species, as shown in Table 3. It should be noted that for some PAI species (e.g., 3D printed) it was not possible to capture the fingerprint with the optical sensor. However, those APs were still included (5) in the database in order to show that this PAI species was considered in the data collection. In a similar manner, the acquisition of particular PAI species was stopped when it turned out that those materials showed no conductive properties. Thus, the focus lay on acquiring APs with measurable impedance values. Hence, non conductive materials were, e.g., additionally coated with electric paint, or, e.g., glycerol was added during the casting process. Those augmentations are listed on the right-hand side of Table 3 and example photos are shown in Figure 5. While both nanotips and bare paint can be applied to solid PAIs, the viscous bare paint cannot be used on softer PAIs without destroying the ridge lines. An overview of the impedance values for all conductive presentations is plotted in the Figure A1 in Appendix A.

The goal was to collect a wide variety of different PAI species in first place. Hence, the used materials were selected based on their availability and experience from former projects. All ingredients are easily purchasable, and their fabrication requires low expertise to allow sufficient numbers of PAIs for the data collection. Based on the results, more sophisticated PAIs exploiting the vulnerability of this capture device can be created.

For the experimental protocol, different dataset partitions were defined in order to evaluate the following scenarios. In general, all partitions were split into non-overlapping training and test sets to guarantee a fair evaluation of the proposed fingerprint PAD method. First, a mixed partitioning was used, where each conductive PAI species was present in both training and test sets. A maximum of 1/3 of APs from a particular PAI species were used for training, and the other samples were seen only during testing. This served as a baseline experiment with only known PAI species in the test set. Second, an additional set of experiments aimed to analyse the fingerprint PAD performance towards unknown PAI species. In this context, a leave-one-out protocol was applied were single PAI species were removed from the training set and only occurred during testing. The LOO partitions were created only for conductive materials or their conductive augmentations, respectively. In other words, one particular PAI species (e.g., playdoh) or all its augmentations (e.g., latex + nanotips and latex + bare paint) were not seen during training. Since the same augmentations were applied to multiple base materials, additional LOO partitions excluded all modifications with bare paint or nanotips, respectively.

The numbers of training and testing samples for the different partitions are summarised in Table 4. An identical number of APs and BPs was chosen for the training partition to prevent bias towards a particular class. Additional samples were moved to the test set for the baseline partition. The LOO partitions removed AP samples after filling the training set. In order to maintain a meaningful training set, a maximum of 15 conductive APs from the same PAI species were included in the training set, and finally, the remaining space was filled with random samples from non-conductive PAI species. Hence, the training set had no bias towards one class (AP or BP), nor towards particular PAI species. Therefore, the experiments allowed fair benchmarking of different LOO partitions and sound conclusions regarding the vulnerability towards unknown attacks.

### 5.2. Metrics

The results of the impedance-based PAD algorithm were analysed with the following metrics defined in ISO/IEC 30107-3 [58]:

- Attack Presentation Classification Error Rate (APCER): percentage of APs incorrectly classified as BPs.
- Bona fide Presentation Classification Error Rate (BPCER): percentage of BPs incorrectly classified as APs.

For this purpose, the detection error tradeoff (DET) visualises the fingerprint PAD performance for all possible decision thresholds. While high security applications require a low APCER, low BPCERs represent convenient use cases. In order to benchmark the different results, two operation points were chosen: (i) detection equal error rate (D-EER), as the point where APCER = BPCER; and (ii) APCER_0.2_, as the APCER for a fixed BPCER of 0.2%, thereby representing a convenient scenario.

### 5.3. Results and Discussion

**Figure 6:**
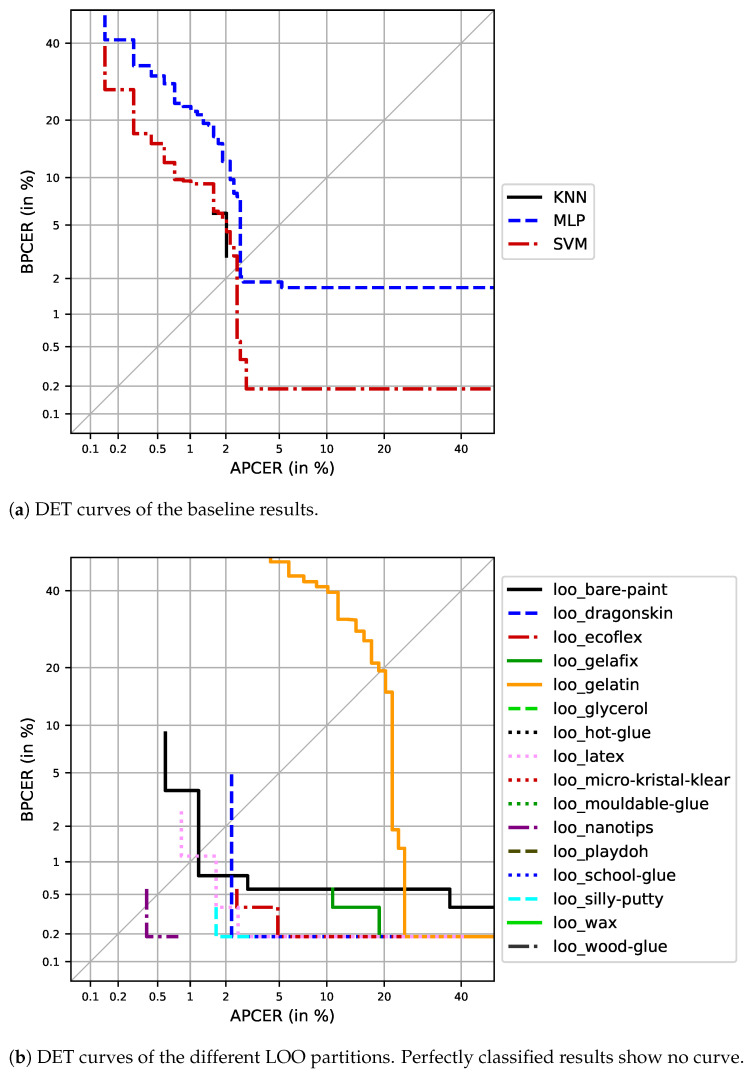
DET plots for the different experiments: (**a**) baseline and (**b**) LOO.

Based on the specified protocol, different experiments were evaluated. The DET plot of the baseline partition is shown in Figure 6a. The SVM approach significantly outperformed the other two approaches; thus, the further analysis and the LOO experiments focused on these results. The plot shows that there is one BP that will always be misclassified as an AP. Hence, a BPCER below 0.2% is not possible for this scenario. On the other hand, over 97% of all APs were correctly classified for both operation points, D-EER and APCER_0.2_.

The next set of experiments evaluated the generalisation capabilities of the fingerprint PAD algorithm towards unknown attacks. The results of the 16 LOO experiments are plotted in Figure 6b. Since the BPs within the test set remained identical to the baseline partition, the same sample kept getting wrongly classified across all LOO partitions. Additionally, the number of APs in the test set was generally lower for the LOO experiments than for the baseline. Thus, the corresponding DET curves ended earlier, and achieving very low APCERs was not possible in these cases. Depending on the PAI species used in the particular training set and their similarity to BPs, the BPCER could be significantly higher than for the baseline partition. On the other hand, some of the conductive PAI species that were left out during training were completely classified as APs without error. Those perfect classifications cannot be shown by the DET plot; thus, the specific error rates in terms of D-EER and APCER_0.2_ are additionally in Table 5 for all experiments.

For the baseline partition, a D-EER of 2.45% and an APCER_0.2_ of 2.89% were achieved. This translates to 20 misclassified APs, which mainly stemmed from the gelatin PAI species. As a consequence, the fingerprint PAD method reliably detected all other known PAI species in the baseline scenario. These findings were basically confirmed by the LOO experiments. For most LOO partitions, a D-EER below 1% was reported, with gelatin being the biggest outlier (D-EER = 19%). It should be noted that the computation of the D-EER anticipated both error rates to be equal at some point. Therefore, the computation reported BPCER/2 for those cases where all APs were correctly classified. Given the one misclassified BP, this resulted in a D-EER of 0.1%. When switching to the convenient threshold for BPCER = 0.2%, additional outliers were revealed. Following gelatin (APCER_0.2_ ≈ 25%), gelafix was misclassified in 7/37 cases (APCER_0.2_ = 19%). However, the least convenient performance was reported after leaving out all bare paint adjustments from training. This LOO group achieved an APCER_0.2_ > 90%, but the D-EER seemed stable at 1.15%. The reason for this behaviour lies in the fact that these unknown attacks have very similar impedance values as the BPs. Hence, the classifier mixed both classes such that the results allowed no separation for very low BPCERs.

Summarising the results, one can say that the concept of using an impedance-based fingerprint PAD module is generally suited for convenient use cases. Except for one constant error, the remaining BPs could be separated from APs and thus kept the false alarm rate low. Additionally, the reported baseline performance showed that a large share of the APs were correctly detected, which indicates strong security against APs in general. However, the following LOO experiments revealed that this fingerprint PAD method is indeed vulnerable against unknown attacks. In particular, PAI species that are conductive by nature (e.g., gelatin and gelafix) pose a severe threat when unknown to the PAD algorithm. Additionally, the results from the LOO bare paint partition show that unknown conductive adjustments are likely to be misclassified as BPs independently of the base material used for the PAI. Since there are more ways to add conductance to PAI species than the ones used in this work, impedance on its own might not be sufficient for secure fingerprint PAD.

### 5.4. Comparison to State-of-the-Art

This Section places our results next to already published ones of state-of-the-art fingerprint PAD approaches. However, since all experimental test sets differ, the focus is on a baseline scenario. Moreover, software-based methods that evaluated the LivDet protocols often only reported the average classification error rate (*ACER*), which is defined as:

(1) $$\begin{array}{c} ACER=\frac{APCER+BPCER}{2} \end{array}$$

Since the error rates highly depend on the database size and the PAI species that are included, those specifications are presented. The main specifications for the comparison are summarised in Table 6. It should be noted that the numbers of PAI species, APs and BPs for the software-based solutions were added up for all evaluated datasets. However, the best performance is usually not achieved through cross-sensor or cross-database experiments; thus, only a fraction of the overall samples were used for this. In general, it is difficult to compare fingerprint PAD performances across totally different scenarios. However, it can be observed that the datasets for software-based methods usually comprised less PAI species than in the data for hardware-based methods. On the other hand, it is possible to evaluate a software-based PAD algorithm with multiple datasets to analyse the generalisation capabilities.

On the other hand, the hardware-based approaches have shown remarkable numbers of PAI species and total database sizes. The reported performances are similar to that of our approach in the baseline scenario. Furthermore, the topic of unknown attacks is addressed by using one-class classifiers. In accordance with our LOO experiments, the overall error rates are generally higher than for the baseline results, which proves the need for further research in this area.

All in all, it can be said that our fingerprint PAD performance is among the state-of-the-art. However, a larger data collection effort is required to confirm these findings regarding the suitability in highly frequented real-world applications.

## 6. Conclusions and Future Work

In this study, an optical capture device was modified to additionally measure the finger’s impedance across nine selected frequencies. These additional data were then used to train an SVM for the task of fingerprint PAD. The idea is that all BPs show a common conductive response, but APs should differ in this regard, due to their physical properties differing from those of human skin. The collected dataset comprises a total of 1672 samples, including 42 different PAI species. The proposed fingerprint PAD method made a real-time decision based on the measured impedance values, thereby taking into account the conductivity itself and the separation between the different frequencies. The experiments showed remarkable results for the baseline scenario, such that an APCER = 2.89% was reached for a convenient use case defined by a BPCER = 0.2%. Hence, 41/42 PAI species were correctly classified in the best case. The only weakness was gelatin APs, which have natural conductance.

On the other hand, the LOO experiments showed additional limitations of this fingerprint PAD method unseen during training: gelafix and bare paint adjustments. While all other PAI species were reliably detected, even if unknown to the classifier, real-life applications can be considered unsafe when there is one attack that succeeds at remaining undetected regularly. Hence, it must be concluded that impedance on its own is not sufficient for secure fingerprint PAD, although all BPs (except for one) were correctly classified.

In this context, it was shown that complementary information channels can be fused to improve the overall PAD performance [47,60]. For this particular capture device, the optical fingerprint image could be used as a second data source, thereby combining software and hardware-based fingerprint PAD approaches. Furthermore, since the display is the illumination source for the optical capture process, differently coloured illuminations are already available, which should allow a multi-spectral fingerprint PAD approach similar to [61,62].

## Figures and Tables

**Table 2:** Nine different frequencies in the range from 1 to 500 kHz are used for the impedance measurements.

	${\mathrm{FQ}}_{1}$	${\mathrm{FQ}}_{2}$	${\mathrm{FQ}}_{3}$	${\mathrm{FQ}}_{4}$	${\mathrm{FQ}}_{5}$	${\mathrm{FQ}}_{6}$	${\mathrm{FQ}}_{7}$	${\mathrm{FQ}}_{8}$	${\mathrm{FQ}}_{9}$
(Hz)	1000	2500	5000	10,000	25,000	50,000	100,000	250,000	500,000

**Table 3:** A detailed list with the number of samples per PAI species.

#	PAI Species	#	Conductive Augmentations
5	3D printed	22	dragonskin + bare paint
17	acryl	22	ecoflex + bare paint
16	dragonskin	20	hot glue + bare paint
32	ecoflex	60	latex + bare paint
18	foil	15	school glue + bare paint
37	gelafix	15	wax + bare paint
69	gelatin	15	wood glue + bare paint
6	glove nitrile		
5	hot glue	23	dragonskin + nanotips
57	latex	19	ecoflex + nanotips
6	micro kristal klear	20	hot glue + nanotips
6	MINT stamp	60	latex + nanotips
20	mouldable glue	20	mouldable glue + nanotips
6	nyloprint	20	school glue + nanotips
8	opti clean	60	silly putty + nanotips
33	paper	15	wax + nanotips
20	playdoh	19	wood glue + nanotips
4	school glue		
27	silicone	13	wood glue + glycerol
20	silly putty		
10	silly putty metallic		
10	wax		
21	windowcolour		
5	wood		
19	wood glue		

**Table 4:** Numbers of samples per dataset partition. The same BPs of the baseline partition were used in all LOO partitions.

	Training	Test
baseline (AP)	223	692
baseline (BP)	223	534
LOO dragonskin ${}^{b,n}$	223	45
LOO ecoflex ${}^{b,n}$	223	41
LOO gelafix	223	37
LOO gelatin	223	69
LOO hot glue ${}^{b,n}$	223	40
LOO latex ${}^{b,n}$	223	120
LOO micro kristal klear	223	6
LOO mouldable glue ${}^{n}$	223	20
LOO playdoh	223	20
LOO school glue ${}^{b,n}$	223	35
LOO silly putty ${}^{n}$	223	60
LOO wax ${}^{b,n}$	223	30
LOO wood glue ${}^{b,n}$	223	34
LOO wood glue glycerol	223	13
LOO bare paint	223	169
LOO nanotips	223	256

*b* = bare paint, *n* = nanotips.

**Table 5:** Results in terms of D-EER and APCER_0.2_ for the particular partitions.

Partition	D-EER	APCER_0.2_	
	(%)	(%)	(#)
baseline	2.45	2.89	20/692
LOO dragonskin	2.24	2.22	1/45
LOO ecoflex	0.28	2.44	1/41
LOO gelafix	0.28	18.92	7/37
LOO gelatin	19.06	24.64	17/69
LOO hot glue	0.00	0.00	0/40
LOO latex	0.98	2.50	3/120
LOO micro kristal klear	0.10	0.00	0/6
LOO mouldable glue	0.10	0.00	0/20
LOO playdoh	0.10	0.00	0/20
LOO school glue	0.10	0.00	0/35
LOO silly putty	0.18	1.67	1/60
LOO wax	0.10	0.00	0/30
LOO wood glue	0.10	0.00	0/34
LOO wood glue glycerol	0.10	0.00	0/13
LOO bare paint	1.15	90.53	153/169
LOO nanotips	0.38	0.39	1/256

**Table 6:** Comparison of state-of-the-art with the proposed fingerprint PAD method regarding the main specifications. The error rates are presented in percentages.

Ref.	APCER	BPCER	ACER	# PAI Species	# APs	# BPs
software-based						
[34]	-	-	1.74	15	≈11,000	≈10,200
[35]	-	-	2.23	13	≈9700	≈9200
[40]	7.30	1.00	4.15	12	≈13,000	≈9500
[44]	8.22	0.20	4.71	12	≈9700	≈5700
hardware-based						
[47]	1.81	0.20	1.01	45	4339	19,711
[57]	5.00	1.11	3.06	7	1386	396
[59]	3.55	0.20	1.83	16	21,700	14,892
one-class + hardware-based						
[41]	50.20	0.20	25.20	12	5531	11,880
[46]	6.59	0.20	3.40	45	4339	19,711
[48]	5.00	3.41	4.21	101	121	233
**ours**	2.89	0.20	1.55	42	915	757

## Data Availability

The data is available for in-house testing during an internship at da/sec (https://dasec.h-da.de, accessed on 20 August 2021).

## Abbreviations

The following abbreviations are used in this manuscript:

ACER	Average Classification Error Rate
AP	Attack Presentation
APCER	Attack Presentation Classification Error Rate
BP	Bona fide Presentation
BPCER	Bona fide Presentation Classification Error Rate
CNN	Convolutional Neural Network
D-EER	Detection Equal Error Rate
DET	Detection Error Tradeoff
GAN	Generative Adversarial Network
GMM	Gaussian Mixture Model
ITO	Indium-Tin-Oxide
KNN	K-nearest Neighbours
LOO	Leave-one-out
MLP	Multi-Layer Perceptron
OCT	Optical Coherence Technology
PAD	Presentation Attack Detection
PAI	Presentation Attack Instrument
SVM	Support Vector Machine
TFT	Thin Film Transistor

## Appendix A

Medians and standard deviations of the impedance values for all conductive presentations across all nine frequencies are plotted in Figure A1.
